# Practical Identifiability of Plant Growth Models: A Unifying Framework and Its Specification for Three Local Indices

**DOI:** 10.34133/plantphenomics.0133

**Published:** 2024-02-09

**Authors:** Jean Velluet, Antonin Della Noce, Véronique Letort

**Affiliations:** MICS Laboratory, CentraleSupelec, Paris-Saclay University, Gif-sur-Yvette, France.

## Abstract

Amid the rise of machine learning models, a substantial portion of plant growth models remains mechanistic, seeking to capture an in-depth understanding of the underlying phenomena governing the system’s dynamics. The development of these models typically involves parameter estimation from experimental data. Ensuring that the estimated parameters align closely with their respective “true” values is crucial since they hold biological interpretation, leading to the challenge of uniqueness in the solutions. Structural identifiability analysis addresses this issue under the assumption of perfect observations of system dynamics, whereas practical identifiability considers limited measurements and the accompanying noise. In the literature, definitions for structural identifiability vary only slightly among authors, whereas the concept and quantification of practical identifiability lack consensus, with several indices coexisting. In this work, we provide a unified framework for studying identifiability, accommodating different definitions that need to be instantiated depending on each application case. In a more applicative second step, we focus on three widely used methods for quantifying practical identifiability: collinearity indices, profile likelihood, and average relative error. We show the limitations of their local versions, and we propose a new risk index built on the profile likelihood-based confidence intervals. We illustrate the usefulness of these concepts for plant growth modeling using a discrete-time individual plant growth model, LNAS, and a continuous-time plant population epidemics model. Through this work, we aim to underline the significance of identifiability analysis as a complement to any parameter estimation study and offer guidance to the modeler.

## Introduction

Identifiability analysis is what helps the design of a parametric model, from its conceptual formalization to the estimation of its parameter using collected data in the field. In plant growth modeling especially, the importance of this analysis for the modeling process varies according to the chosen modeling paradigm. In our paper, the term “growth model” is employed broadly, encompassing models that address both the biological aspect of growth (quantifiable changes such as biomass and yield) and the developmental aspect of plants (structural and morphological changes of the plant’s organs. Two main kinds of modeling approaches can be distinguished [1]: (a) Mechanistic models (also known as conceptual, knowledge-driven, or white box models) are grounded in the underlying biological principles and physical laws of the system, and aim to provide a detailed understanding of the mechanisms involved in plant growth. The different terms appearing in mechanistic models’ equations have biological interpretations. (b) Empirical models (also called descriptive, statistical, data-driven, or black box models) rely on large datasets, and their construction consists in choosing an a priori flexible model structure (from the simplest, linear models, to the recent popular neural networks) whose parameters are estimated using a training dataset and do not necessarily have an interpretation (they generally do not have one): The model is designed as a “black box” able to faithfully reproduce some input–output relationship. No expert in plant growth is a priori required there. With the spread of inexpensive and efficient sensors, machine learning and deep learning models have gained popularity in plant growth modeling, particularly for applications involving large-scale data analysis, classification, and prediction [2]. However, these models have limitations in terms of interpretability and generalizability, as they cannot be extrapolated to conditions not included in their training dataset.

Therefore, despite the highly mediatized rise of machine learning algorithms, mechanistic approaches still have an important place in plant growth modeling. They can help assess new management adaptations and unmeasured environmental impacts [3,4], and guide the breeding of more resilient crops [5]. They are even irreplaceable as soon as experimental data are scarce and costly. According to [6], mechanistic models can be roughly sorted into two categories.

(a) Complex models consisting of many interacting submodules at the finest scales, which aim at describing every single process of plant development and physiology (e.g., [7,8]) but still limited in their scalability [3,9]. In this approach, the parameter values are set directly from experimental measurements or from the bibliography, and the model is evaluated on its ability to present emergent properties that reproduce observed behaviors not explicitly described in the model. Parameter estimation by global inversion of the model is avoided here since it would introduce potential compensations between different model components.

(b) Models aiming at describing the plant behavior in a more global way with only a limited set of main equations and as few parameters as possible (e.g., GreenLab [10], LNAS [11]). This approach is in fact partly empirical since, most often, some model components include parameters that are unknown or cannot be directly measured, hence the need to estimate them from data on the real system or on its subsystems, using a statistical inference procedure [12]. Since these parameters have biological meanings, their estimated values are expected to be as close as possible to their respective “true” values, which raises the problem of the uniqueness of the solutions of the procedure. We call identifiability analysis the study of the correspondence between the parameters of a model and its outputs. A lack of identifiability does not necessarily imply that the parameter estimation techniques will fail but that some obtained numerical estimates might be meaningless [13].

There are two kinds of identifiability analyses. Structural identifiability [14] analysis aims at determining whether the function associating the parameters to the model outputs is one-to-one. Here, the observable variables of the system are assumed to be perfectly measured, with infinite resolution and acquisition duration if wished ([15,16,17]). Structural identifiability analysis consists indeed in exploring the model structure in itself. Proving nonidentifiability can lead the modelers to change specific aspects of their models, e.g., change the domain where the parameters are searched, or change the expression of the model.

The definitions found in the literature for structural identifiability are generally consistent, but they might be more or less specific depending on the authors [18,19]. Several methods have been proposed for structural identifiability analysis of a model, using power series expansion, Lie groups, differential algebra, and differential geometry, as detailed in [15] or [20], among others. Analytical proofs are often impeded by the complexity of the symbolic calculations that can become intractable even when the model is relatively simple [21]. Numerous software tools have been developed to automatically perform this task, starting from precursors like DAISY, released in 2007 [22]), to more recent ones such as the Python module StrikePy [23], the Julia package StructuralIdentifiability [24], or the Matlab toolbox RORC-DF [25], all of which were released in 2022. Rey Barreiro and Villaverde [26] benchmarked 13 different tools for global identifiability analysis and reported that the Maple toolbox SIAN [27] and the Julia package StructuralIdentifiability [24] outperformed the others in terms of reliability and execution speed.

The second kind of identifiability analysis, namely, practical identifiability [28] consists of determining the accuracy of the estimated parameters, given an observation protocol of the system under study. Here, measurement errors, uncertainties, and limited resolution of data acquisition are considered. As for its quantification, the situation is more confused than for structural identifiability since multiple concepts coexist and may not necessarily be consistent.

Among others, some indices use the likelihood geometry (profiled or not) to quantify its flatness [29], others measure the mean error of the estimator [18] and assess the full rank of a sensitivity matrix [30], and a wide range of indices are based on the Fisher information matrix, also considered for the optimization of experimental design [28]. Some authors refer to practical identifiability as a posteriori identifiability, highlighting the fact that this analysis can be conducted after the data acquisition, while structural identifiability is termed a priori identifiability, emphasizing that it depends solely on the model itself (e.g., in [19,26,31]). However, in real-life applications, practical identifiability can also be performed before data collection, and it is recommended to do so as it provides valuable information on the required accuracy of measurements, minimal time, or spatial resolution. Therefore, we prefer here to use the terms structural and practical.

In this context, our objective is, first, to show how several versions of structural and practical identifiability definitions can be gathered under a common general formalism that needs to be instantiated depending on each application case. In a more applicative second step, we focus on three widely used indices for quantifying practical identifiability, namely, the collinearity indices [32], profile likelihood-based confidence intervals [29] and average relative error (ARE) [18]. We highlight the drawbacks of the local versions and propose some extensions. We also propose a new risk index, built on the profile likelihood-based confidence intervals. We illustrate our methodology by analyzing the practical identifiability of two dynamical systems: a discrete-time plant growth model and a continuous-time epidemics model in plant populations. For the sake of clarity, the descriptions of the two models are switched to the “Applications and Results” section.

## Identifiability Definitions

### Model formalism

In this work, we consider the problem of identifiability of continuous-time and discrete-time dynamic models, which represent the majority of plant growth models. Within this framework, the system (e.g., the plant individual or population) is assumed to be fully characterized by a set of *m* variables forming a state vector *x*(*t*) ∈ *ℝ^m^* that can evolve with time *t* ∈ *ℝ*_+_ from an initial vector *x*_0_ under the influence of *d* control or input variables *u*: *ℝ*_+_ → *ℝ^d^*. It can occur that only a subset of input trajectories can be possibly applied to the system: we note U the set of admissible input vectors. The set of possible vector values reached by *x*(*t*) for any admissible values of *x*_0_ and *u*(*t*) is denoted $X\subset {\mathbb{R}}^{m}$ and, for simplicity’s sake, assumed Euclidean. The state variables drive the dynamics of the system, but they are not necessarily all observable. An observation function *g* defines which quantities *y*(*t*) ∈ *ℝ^n^* can be considered accessible. Such a model can thus be generically described by the following system:

$$S_{\theta }:\left\{\begin{array}{c} \forall t\in {\mathbb{R}}_{+},\;\dot{x}(t)=f(x,\theta ,u,t),\\ x(0)=x_{0}\\ \forall t\in {\mathbb{R}}_{+},\;y(t)=g(x,\theta ,u,t) \end{array}\right.$$ (1)

where the evolution and observation functions, f:X×Θ×$U\times {\mathbb{R}}_{+}\to X$ and $g:X\times \Theta \times U\times {\mathbb{R}}_{+}\to Y$, depend on a parameter vector *θ* ∈ Θ, assumed to be an open and connected subset of *ℝ^p^*. The discrete-time formalism can be deduced by changing *t* ∈ *ℝ*_+_ by *t* ∈ *ℕ* and by replacing the differential equations with difference equations. It will not be detailed here; for more details, see [33].

### Structural identifiability: Generalization of its definition

Structural identifiability is concerned with whether the parameter *θ* can be uniquely determined by a given relationship between the observable model outputs *y* and inputs [16]. We will use the notation *t* ∈ *ℝ*_+_ ↦ *x*(*t*; *x*_0_, *θ*, *u*) to denote the solution of the differential q. 1 verified by *x* with initial condition *x*_0_, parameter *θ*, and control *u*. For a chosen set of observable variables *y*, the model is said to be structurally identifiable if simulations performed with two different parameter vectors generate different observables *y*. A more formal definition can be adapted from [34]:

**Definition 1:** Let $X_{0}\subset X$ be a nonempty subset of initial conditions. Let $U_{0}\subset U$ be a nonempty subset of admissible controls. We say that parameter *θ_i_* is structurally locally identifiable for initial condition if, for all *θ* ∈ Θ, there exists a neighborhood $V_{\theta }$ of *θ* such that

$$\begin{array}{c} \forall \theta '\in V_{\theta },\forall x_{0}\in X_{0},\forall u\in U_{0},\\ \left(\forall t\in R_{+},\;g(x(t;x_{0},\theta ,u),\theta ,u,t)=g(x(t;x_{0},\theta ',u),\theta ',u,t)\right)\Rightarrow \theta_{i}=\theta {'}_{i} \end{array}$$ (2)

The parameter *θ_i_* is said structurally globally identifiable on Θ if $V_{\theta }$ can be chosen as Θ for all *θ* ∈ Θ. Note that in certain definitions, such as the one proposed by [16], the implication in Eq. 2 needs to be verified for “generic” *θ* only, i.e., for all *θ* ∈ Θ except for a subset of a closed set of Lebesgue measure zero. However, we have observed that for real-world applications, researchers might be theoretically interested in obtaining a comprehensive understanding of their model’s behavior in terms of identifiability as well with regard to potential nonidentifiabilities occurring on a negligible subset.

A model is structurally globally identifiable if all its parameters are structurally globally identifiable.

The different definitions of local structural identifiability reviewed in [19] can be encompassed as particular cases of Definition 1. Tunali and Tarn [35] introduced the concept of *x*_0_-identifiability (applied in [36], for example), which corresponds to the case where $X_{0}=\left\{x_{0}\right\}$. Surprisingly enough, no symmetric definition has been proposed to characterize the notion of *u*-identifiability ($U_{0}=\left\{u\right\}$). However, in their benchmark, Rey Barreiro and Villaverde [26] distinguished cases where the input is known or not, which is related to such notion. Hong et al. [37] proposed a definition of global identifiability where Definition 1 has to be true for every admissible input vector, which corresponds to the case where $U_{0}=U$. This definition could be applied to cases where the input vector is not under control. For example, if the system is a plant growing outdoors, the input vector can represent solar exposition, precipitation, temperature, etc. The question of interest is whether the parameters can be uniquely estimated from given observations regardless of the past conditions the plant experienced. In contrast, one might be interested in investigating whether one particular input condition *u* could be applied that would make the model identifiable: the definition should in that case hold for one specific *u*. For example, if we consider a plant growing in a greenhouse where we have total control over the outside conditions, then it is sufficient for Definition 1 to hold for a particular control vector of interest. Identifiability analysis can thus lead to optimal design of experiments, the objective being here to achieve the highest accuracy in parameter estimation rather than focusing on the plant growth itself.

Definition 1 points out that structural identifiability always boils down to the injectivity of some function defined over Θ and taking values in the functional space $M≔C^{0}(X_{0}\times U_{0},C^{0}({\mathbb{R}}_{+},Y))$, where Y is the output space of the observation function *g*. The model function $M:\theta \in \Theta \mapsto M\left(\theta \right)\in M$ gives a collection of trajectories that are indexed by the initial condition chosen in $X_{0}$ and the control chosen in $U_{0}$. Using this notation, Definition 1 is equivalent to the following statement: Let M:Θ→M be the function associated with the model. We say that parameter *θ_i_* is locally structurally identifiable if for all *θ* ∈ Θ there exists a neighborhood $V_{\theta }$ such that for all $\theta^{\prime }\in V_{\theta }$, *M*(*θ*) = *M*(*θ*′) ⇒ *θ_i_* = *θ*′*_i_*, where *θ_i_* is the *i^th^* component of the parameter vector *θ*.

*M*(*θ*) represents a function mapping a set of parameters in space Θ to an output space M, which must be specified according to the modeler’s objectives. The output space of the model M is the space of continuous mapping associating an initial condition to a continuous trajectory in the observed state space Y. We can even consider situations where time is discrete, corresponding to an output space being a collection of sequences, or situations where the dynamics are stochastic, corresponding to an output space containing process distributions.

Thus, this definition has the advantage of gathering many existing ones by choosing appropriately the set M. Each choice of *M* and M can be useful for a particular application, and this generic formulation encourages authors to better specify their definition of identifiability.

### Practical identifiability: General concept

While structural identifiability is concerned with the injectivity of the model with respect to its parameters, practical identifiability addresses different questions, which are related with the ability to identify the parameters by statistical inference on a set of experimental observations. One of the main difficulties in defining practical identifiability is the large variety of definitions one can find in the literature. In this section, we introduce the theoretical framework to compare the stakes and objectives of those various definitions.

A first aspect of practical identifiability is related to the way the system is observed and how observing it provides information on the model configuration. It is implicitly linked with the notion of observation protocol, also referred to as experimental plan in the literature. An observation protocol can be formalized as a function associating a trajectory m∈M of the system to a collection of data or measurements, belonging to a finite-dimensional space. A natural example of observation protocol is simply to evaluate the trajectory at specific initial condition $x_{0}\in X_{0}$, specific control $u\in U_{0}$, and specific observation times *t*_1_, …, *t_T_*. The function associated with the observation has the following expression:

$$\mathrm{Obs}:m\in M\mapsto (m(x_{0},u,t_{1}),\ldots ,m(x_{0},u,t_{T}))\in Y^{T}.$$ (3)

A definition of practical identifiability studies the properties of the function $\theta \in \Theta \mapsto \mathrm{Obs}\left(M\left(\theta \right)\right)\in Y^{T}$ by computing its collinearity indices [32]. This definition of practical identifiability constitutes some sort of restriction of the structural identifiability analysis to a finite-dimensional output space. The collinearity indices are discussed with further details in the next section.

In a more realistic situation, the observation protocol is tainted by measurement error, e.g., due to the accuracy of the devices used in the experiments, such that one can have two different observations of the same trajectory. As a consequence, the observation protocol can be formalized in a more generic way as a function associating a trajectory to the probability distribution of the observations on this trajectory. This leads to the following generic expression of the observation function:

$$\mathrm{Obs}:m\in M\mapsto \mathrm{Obs}\left[m\right]\left(dz\right)\in P\left(Z\right),$$ (4)

where Z is a finite-dimensional space containing the output of the observation protocol (one dimension for each measurement). For instance, we can assume that the trajectory in q. 3 is observed with an additive Gaussian noise $N\left(0,\Sigma \right)$, which leads to the following expression:

$$\begin{array}{c} \mathrm{Obs}:m\in M\mapsto N(m(x_{0},u,t_{1}),\Sigma )⊗\ldots ⊗N(m(x_{0},u,t_{T}),\Sigma )\in P\left(Z\right). \end{array}$$ (5)

The deterministic case in Eq. 3 can also be rewritten in this framework using Dirac distributions:

$$\mathrm{Obs}:m\in M\mapsto \delta \left[m(x_{0},u,t_{1})\right]⊗\ldots ⊗\delta \left[m(x_{0},u,t_{T})\right].$$ (6)

In the case where there exists a *σ*-finite measure *λ* defined over Z such that for all *θ* ∈ Θ the distribution Obs[*M*(*θ*)] is absolutely continuous with respect to *λ*, then we can consider the density $z\in Z\mapsto p\left(z;\theta \right)\in {\mathbb{R}}_{+}$ for all *θ* ∈ Θ. The density *p*(*z*; *θ*) is exactly the density of the observations. Another definition of practical identifiability, due to [29], is based on the analysis of the likelihood function *θ* ↦ *p*(*z*; *θ*) for a fixed set of observations *z*. This definition is further discussed in the “Profiled likelihood-based risk index” section.

Other definitions of practical identifiability are implicitly linked with the parameter estimation methodology. Parameter estimation is divided into two main paradigms: deterministic approaches and stochastic approaches. Deterministic approaches consist in maximizing either likelihood or a posterior distribution, while stochastic approaches consist in either determining the posterior distribution of the parameters with respect to a given prior or sampling from this posterior distribution. Overall, all these estimation procedures can be formalized by a function associating a set of observations *z* to a probability distribution over Θ

$$z\in Z\mapsto \hat{\mu }(d\theta ;z)\in P\left(\Theta \right).$$ (7)

Indeed, the case where the estimate $\hat{\theta }\left(z\right)$ is a deterministic function of *z* is represented by a Dirac distribution $\delta \left[\hat{\theta }\left(z\right)\right]\in P\left(\Theta \right)$. When the estimation process involves stochasticity, $\hat{\mu }\left(d\theta ;z\right)$ can represent various distributions. It could be the distribution of estimated parameter $\hat{\theta }$ based on the observed data *z*. Alternatively, if a Bayesian approach is taken, it could represent the posterior distribution of *θ*, denoted as *μ*(d*θ*| *z*). Finally, if a Markov chain Monte-Carlo (MCMC) is used to approximate this posterior, $\hat{\mu }\left(d\theta ;z\right)$ would be the empirical distribution derived from the MCMC samples. Another definition of practical identifiability uses the ARE comparing the resulting distribution of the estimate, which is $\int_{Z}\;‍\hat{\mu }\left(d\theta ;z\right)\mathrm{Obs}\left[M\left(\theta \right)\right]\left(dz\right)$, with the Dirac distribution *δ*[*θ*] centered at the true value of the parameter *θ*. This definition of practical identifiability is further discussed in the “Average relative error” section.

### The particular case of collinearity indices: Practical identifiability in the absence of measurement noise

It can be of interest, in the initial phase of the analysis of practical identifiability, to evaluate the observation protocol Obs without the interference of measurement errors. By considering an ideal experimental protocol, we gain insights into the influence of its design (number, type and times of measures, etc.) on parameter identification.

In the absence of measurement noise, the distribution of the observations associated with the observation protocol Obs(*M*(*θ*)) is deterministic, i.e., a Dirac distribution centered at the values of the observables at the considered times. For instance, if the experimental protocol consists of observing the system at times *t*_1_, *t*_2_, …, *t_T_*, the distribution of the observations is

$$\mathrm{Obs}(M(\theta ))(\mathrm{dy})=\delta [(y(t_{1},\theta ),\ldots ,y(t_{T},\theta )](\mathrm{dy})$$ (8)

We can therefore consider the function η:Θ→Y such that for all *θ* ∈ Θ, Obs(*M*(*θ*)) = *δ*[*η*(*θ*)], and study its properties to derive information on the identifiability of the system.

If the experimental protocol is well designed, the injectivity of the function *η* can be equivalent to the structural identifiability of the model. Conversely, the study of the function *η* can help to diagnose potential structural nonidentifiability sources, which result from compensation effects between parameters. A compensation effect of order *k* means that a variation in a parameter can be compensated by variations in some *k* − 1 other parameters to get an unchanged model output. Most methods for detecting and exhibiting such local compensation effects are based on the sensitivity matrix (see below) and the Fisher information matrix [31,38]. In contrast, the method proposed by Hengl et al. [39] consists of performing numerous fittings from different initial guesses and investigating nonparametrically (using the alternating conditional expectation method) whether the obtained parameter set forms a low-dimensional manifold. This approach can detect nonlinear dependencies between the parameters, but it comes with a computational cost. In our study, we consider the method of collinearity indices introduced in [32], which is relatively easy to compute even for complex models and can deal with large parameter sets (see also [40] for its application to large-scale models, i.e., with a large number of parameters). We briefly recall the method here. The model output is linearized around a reference parameter *θ*^∗^: $\forall \theta \in V_{\theta^{*}}$ a neighborhood of *θ*^∗^,

$$\eta \left(\theta \right)\approx \eta \left(\theta^{*}\right)+{\left.\frac{\partial \eta }{\partial \theta }\right|}_{\theta =\theta^{*}}\left(\theta -\theta^{*}\right)$$

and we define $S≔{\left(\frac{\Delta \theta_{j}}{SC_{i}}\frac{\partial \eta_{i}}{\partial \theta_{j}}\left(\theta^{*}\right)\right)}_{i,j}$, an *m* × *n* matrix where *m* is the number of observations, *n* is the number of parameters, Δ*θ_j_* is the uncertainty range of parameter *j*, and *SC_i_* is a normalization constant for the observables (usually the mean). Although rarely noninvertible, the positive semi-definite matrix *S^T^S* can be ill conditioned. The collinearity index of the parameter subset *K* ⊂ [[ 1,*m*]] is defined as $\gamma_{K}≔\frac{1}{\sqrt{\lambda_{K}}}$, where *λ_K_* is the smallest eigenvalue of the matrix obtained from *S^T^S* by keeping only the columns corresponding to the parameters in *K* and normalizing them to 1. By convention, *γ_K_* ≔  + ∞ if *λ_k_* = 0.

The interpretation of the collinearity index is simple: A change in the output vector *η*(*θ*) caused by a shift of a parameter *θ_j_* ∈ *K* can be compensated up to $\left(1-\frac{1}{\gamma_{K}}\right)\cdot 100\%$ by shifting the other parameters in the subset *K*. If there is a large but noninfinite collinearity index for a subset of parameters, the model is stricto-sensu identifiable, but when we try to estimate the parameters with noisy data, we may find that the parameter cannot be estimated with a satisfactory confidence interval due to large compensation effects. Collinearity indices are somehow close to structural identifiability indicators since they consider no measurement noise. However, they depend on the number and times of measurements and, as such, they do not consider the measures as ideal with infinite resolution, which classifies them into the practical identifiability methods.

Collinearity indices can be thought of as a quantification of the local and practical identifiability of the model parameters. If the matrix *S^T^S* is singular, then there exists *θ*′ in a neighborhood of *θ* and distinct from *θ* such that *η*(*θ*) = *η*(*θ*′). Otherwise, collinearity indices quantify how close the output function *η* is to being locally noninvertible (high values of collinearity indices implying that the matrix is ill conditioned).

The application of the local collinearity indices method is illustrated on the LNAS model in the “LNAS, a simple plant growth model” section.

### Profiled likelihood-based risk index

Let us consider the particular case of a dynamical system *S* observed with an additive Gaussian error at *T* observation times *t*_1_, …, *t_T_*. For practical identifiability, we define:

$$\forall \theta \in \Theta ,\chi^{2}\left(\theta \right)≔\sum_{k=1}^{m}\sum_{l=1}^{d}\frac{{\left(Y_{kl}-Y_{k}\left(x_{\theta },\theta ,t_{l}\right)\right)}^{2}}{\left(\sigma_{\mathrm{kl}}^{D}\right)2}$$ (9)

where *y_kl_* denotes data points for each observable *k*, measured at time points *t_l_* and $\sigma_{\mathrm{kl}}^{D}$ the corresponding measurement error. An interesting quantity is the profile likelihood for the *i*th parameter *θ_i_* as $\chi_{\mathrm{PL}}^{2}\left(\theta_{i}\right)=\underset{{\left(\theta_{j}\right)}_{j\neq i}}{\mathrm{Min}}\left[\chi^{2}\left(\theta \right)\right]$. In the case where Θ = *ℝ*^dim(*θ*)^, Raue et al. [29] introduced the profile likelihood-based confidence interval (PLCI) at level *α* for the parameter *θ_i_* by $\left(\theta_{i}^{min}\left(\alpha \right),\theta_{i}^{max}\left(\alpha \right)\right)\;$, where

$$\begin{array}{ll} & \theta_{i}^{\min}\left(\alpha \right)=\underset{\;t\in {\mathbb{R}}_{+}}{\inf}\left\{\theta_{i}^{*}-t:\theta^{*}-te_{i}\in \Theta ,\chi_{\mathrm{PL}}^{2}\left(\theta^{*}-te_{i}\right)-\chi_{\mathrm{PL}}^{2}\left(\theta^{*}\right)\geq \Delta \alpha \right\}\\ & \theta_{i}^{\max}\left(\alpha \right)=\underset{\;t\in {\mathbb{R}}_{+}}{\inf}\left\{\theta_{i}^{*}+t:\theta^{*}+te_{i}\in \Theta ,\chi_{\mathrm{PL}}^{2}\left(\theta^{*}+te_{i}\right)-\chi_{\mathrm{PL}}^{2}\left(\theta^{*}\right)\geq \Delta \alpha \right\} \end{array}$$

with Δ*α* = *χ*^2^(*α*, 1) the *α*-quantile of *χ*^2^ and *e_i_* is the *i*th vector of the canonical basis of *ℝ*^dim(Θ)^. Computing the PLCI can be numerically expensive, but a method can be found in [41], relying on a modified Newton–Raphson iteration to solve a system of equations that defines its endpoints [considering that at these points, the gradient *∂χ*^2^(*θ*)/*∂θ*_∼*i*_ = 0, where *θ*_∼*i*_ = (*θ*_1_, …, *θ*_*i*−1_, *θ*_*i*+1_, …, *θ_p_*)]. Raue et al. define a practically nonidentifiable parameter as follows.

**Definition:** Let *α* ∈ [0; 1). The component *θ_i_* of parameter *θ* is said to be practically unidentifiable at level *α* if the PLCI $\left(\theta_{i}^{\min}\left(\alpha \right);\theta_{i}^{\max}\left(\alpha \right)\right)$ has an infinite length: $\lambda \left(\left(\theta_{i}^{\min}\left(\alpha \right);\theta_{i}^{\max}\left(\alpha \right)\right)\right)=+\infty$.

Note that this situation of practical nonidentifiability can occur even if the objective function has a unique minimum for this parameter (case of structural identifiability). In the case where the parameters have biological meanings and, therefore, naturally restricted to certain a priori interval, they should be first mapped to *ℝ* via a bijective function (e.g., natural logarithm for parameters that must remain positive) before applying Raue’s PLCI method.

An important limitation of this index is that the binary answer to the problem of practical identifiability is not informative enough because it lacks some quantification: It does not allow to distinguish cases where the nonidentifiability could be easily eliminated (by reducing the noise, for instance) from cases where they are irremediable (case of structural nonidentifiability for instance).

To overcome this issue, we propose an extension of Raue’s PLCI method by quantifying the smallest *α* level such that the PLCI remains finite.

**Definition:** Let *θ_i_* ∈ Θ*_i_* ⊂ *ℝ*. We define a profiled likelihood-based risk index *r_i_* as:

$$\begin{array}{c} \mathrm{ri}=\frac{1}{\text{sup}\left\{\alpha \in \left(0,1\right):\lambda \left(\left(\theta_{i}^{\min}\left(\alpha \right);\theta_{i}^{\max}\left(\alpha \right)\right)\right)<+\infty \right\}} \end{array}$$ (10)

A risk index close to 1 can be interpreted as a parameter having a good level of practical identifiability. Besides bringing a quantification of practical identifiability, this definition has two side advantages: (a) It takes into account the information brought by a dataset compared to the a priori information Θ*_i_* on each parameter, and (b) it includes the case where the set $\left\{\theta_{i}\in \Theta ,|,\chi_{\mathrm{PL}}^{2}\left(\theta \right)<\Delta_{\alpha }\right\}$ is not connected, i.e., with a bimodal profiled likelihood (with two minima separated by a “hill”) and with two distinct regions such that $\chi_{\mathrm{PL}}^{2}\left(\theta \right)<\Delta_{\alpha }$.

### Average relative error

Another widely used measure of practical nonidentifiability, introduced in [18], is ARE indices. The ARE of parameter *θ_i_*, estimated with the method associated with the estimator distribution $\hat{\mu }\left(d\theta ;z\right)$, is

$${\mathrm{ARE}}_{i}=\frac{1}{|\theta_{i}^{*}|}\iint_{Z\times \Theta_{i}}‍|\theta_{i}^{*}-\theta_{i}|{\hat{\mu }}_{i}(d\theta_{i};z)\mathrm{Obs}\left[M\left(\theta^{*}\right)\right]\left(dz\right),$$ (11)

where ${\hat{\mu }}_{i}\left(d\theta_{i};z\right)$ is the marginal distribution of the *i*th component of estimator $\hat{\theta }$. When $\theta_{i}^{*}=0$, which can be the case in the context of variable selection for instance, we can rather consider the expected absolute value of the estimator, i.e.

$$\begin{array}{c} AE_{i}=\iint_{Z\times \Theta_{i}}‍|\theta_{i}|{\hat{\mu }}_{i}(d\theta_{i};z)\mathrm{Obs}\left[M\left(\theta^{*}\right)\right]\left(dz\right) \end{array}$$ (12)

These errors are computed in the following way: *N* sets of model outputs *z*^(*k*)^ are generated for which we estimate ${\hat{\theta }}^{\left(k\right)}$, *k* ∈ {1, …, *N*}. The *ARE* index of parameter *θ_i_* is defined by

$${\mathrm{ARE}}_{i}=100\times \frac{1}{N}\sum_{k=1}^{N}‍\frac{|\theta_{i}^{*}-{\hat{\theta_{i}}}^{\left(k\right)}|}{|\theta_{i}^{*}|}$$ (13)

The smaller the parameter estimation errors, the more identifiable the parameters are and therefore the closer the parameter’s ARE index is to 0. This index reflects the average uncertainty on the estimated parameters. For their interpretation and practical use, it is important to remember that ARE values are dependent on the estimation procedure (initial guess and optimization algorithm) and very specific to the reference parameter *θ*^∗^.

Regardless of how computationally expensive it is, we propose to “globalize” the ARE indices by computing their mean when the parameters *θ*^∗^ are drawn from a given prior distribution.

## Applications and Results

We performed two case studies to illustrate the application of these concepts: a discrete-time individual plant growth model, LNAS, which will allow exploring the use of the collinearity indices for detecting compensation effects, and a continuous-time plant population epidemics model, which will be used to investigate the interest of switching from local indices to their global extensions.

### LNAS, a simple plant growth model

#### Brief presentation of LNAS

LNAS belongs to the family of predictive plant growth models that have a lower level of complexity than descriptive models because they are mainly used to predict yield or biomass production [11]. Developed to simulate the growth of sugar beet, LNAS is an empirical compartment-scale model aiming at predicting biomass allocation to root and leaf compartments. Biomass production *Q*(*t*) (in *g*. *m*^−2^) at day *t* is modeled using an extension of the Beer–Lambert law, depending on the fraction of intercepted radiation with an extinction coefficient *k_B_* and a parameter of radiation use efficiency *RUE* (Eq. 14):

$$\left\{\begin{array}{cc} \tau \left(t+1\right)= & \;\tau \left(t\right)+\text{max}\left(0,\;T(t)-T_{b}\right)\\ \;Q_{g}\left(t\right)= & \;(1-G_{s}(\tau (t)-\tau_{sen};\mu_{s},\sigma_{s}))\cdot Q_{l}\left(t\right)\\ \;\gamma \left(t\right)= & \;\gamma_{0}+\left(\gamma_{f}-\gamma_{0}\right)\cdot G_{a}(\tau (t);\mu_{a},\sigma_{a})\\ \;Q\left(t\right)= & 0.95\cdot \mathrm{RUE}\cdot \mathrm{PAR}\left(t\right)\cdot \left(1-e^{-k_{B}\frac{Q_{g}\left(t\right)}{e_{g}}}\right)\\ Q_{l}\left(t+1\right)= & \;Q_{l}\left(t\right)+\gamma \left(t\right)\cdot Q\left(t\right)\\ Q_{r}\left(t+1\right)= & \;Q_{r}\left(t\right)+\left(1-\gamma \left(t\right)\right)\cdot Q\left(t\right) \end{array}\right.$$ (14)

*Q_g_*(*t*) is the dry matter of green leaves (in *g*. *m*^−2^) at day *t*, which is converted into a surface via a parameter *eg*. This green surface can intercept the incident photosynthetically active radiation *PAR(t)* received on day *t*, of which a maximum proportion of 95% can be absorbed [42]. *Q_l_*(*t*) and *Q_r_*(*t*) are respectively the total mass of leaves and of roots. A proportion *γ*(*t*) of the biomass production is allocated to the leaf compartment at each time step *t*, modeled as a function of *G_a_* the cumulative distribution of a log-normal (LN) law with median *μ_a_* and standard deviation *σ_a_*. Leaf senescence is similarly modeled as a function of *G_s_* = *LN*(*μ_s_*, *σ_s_*). *τ*(*t*) denotes the thermal time at day *t*. The model thus has only 8 parameters: *θ* = (*RUE*, *e_g_*, *μ_a_*, *σ_a_*, *μ_s_*, *σ_s_*, *γ*_0_, *γ_f_*). The input vector *PAR*, representing the active radiation (in *MJ*/*m*^2^), is assumed to be known as well as the initial biomass (seed mass). The initial mass of dry green leaves and the initial yield are zero.

#### Identifiability results

**Fig. 1.**
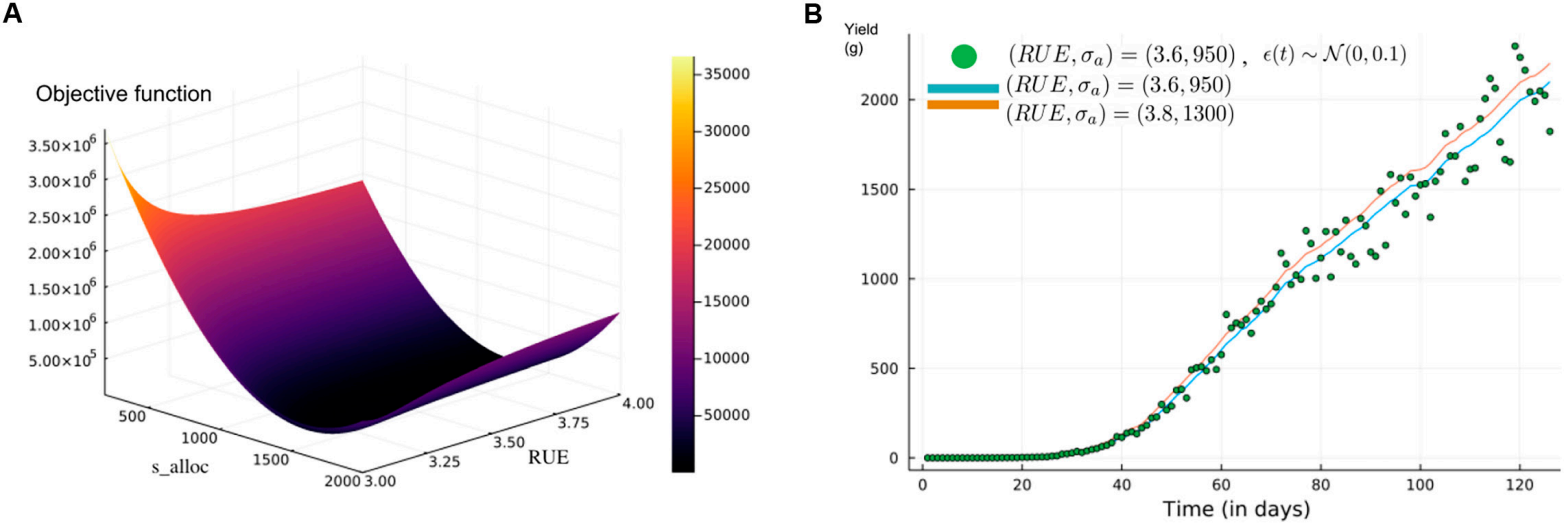
Illustration of the lack of identifiability caused by compensations between two parameters of the LNAS model. (A) Objective function on the grid (*σ_alloc_*, *rue*). (B) Dynamics of the yield (root compartment mass) for (*rue*, *σ_a_*) = (3.6,950) (blue curve) versus for (*rue*, *σ_a_*) = (3.8,1300) (orange), and the virtual noisy data (dots).

The output space is $M≔C^{0}\left(X_{0}\times U_{0},C^{0}\left({\mathbb{R}}_{+},Y\right)\right)$, with $X_{0}$ and $U_{0}$ defined as singletons, as explained in the “Structural identifiability: Generalization of its definition” section. We suppose *x*_0_ to be known, and we observe the dry green leaf biomass and yield every day between 25 and 150. First, we simulated our virtual observables *y_obs_* with an additive Gaussian observation noise $\epsilon \left(t\right)∼N\left(0,0.1\right)$. Then, we computed the collinearity indices for every pair of parameters (Table 1). They allow us to highlight the compensation effects between the parameters [32], hence investigating which pairs of parameters are the most difficult to estimate together. We found that the parameter pair with the highest collinearity index is (*σ_a_*, *RUE*) with a value of about 15, meaning that a small change in *σ_a_* can be compensated up to (1 − 1/15) · 100% = 93% in the outputs by a change in *RUE*. We investigated this result by displaying the three-dimensional (3D) graph of the objective function *χ*^2^ with respect to these two parameters (Fig. 1A). One can indeed note a kind of flat “valley,” symptomatic of a problem of practical identifiability, due to the strong interaction between these two parameters.

**Table 1.** Collinearity indices for every parameter pair of the LNAS model

par.	rue	*e_g_*	*γ*_0_	*γ_f_*	*μ_a_*	*σ_a_*	*μ_s_*	*σ_s_*
rue	∞							
*e_g_*	5.54	∞						
*γ*_0_	2.91	5.87	∞					
*γ_f_*	5.67	2.83	1.96	∞				
*μ_a_*	6.02	3.03	2.03	9.28	∞			
*σ_a_*	15.42	4.10	2.48	8.86	7.90	∞		
*μ_s_*	7.81	3.40	2.20	9.67	10.73	11.28	∞	
*σ_s_*	12.31	5.17	2.79	4.94	6.57	8.74	8.00	∞

To further illustrate this effect, we draw in Fig. 1B the dynamics of the yield (root compartment mass) as a function of time for (*rue*, *σ_a_*) = (3.6,950) (blue curve) versus for (*rue*, *σ_a_*) = (3.8,1300) (orange). These values were arbitrarily chosen by manually observing the heat map of the objective function. Despite these parameter values being respectively 5% and 30% different, the two curves are nearly indistinguishable, both being consistent with the data (dots).

**Fig. 2.**
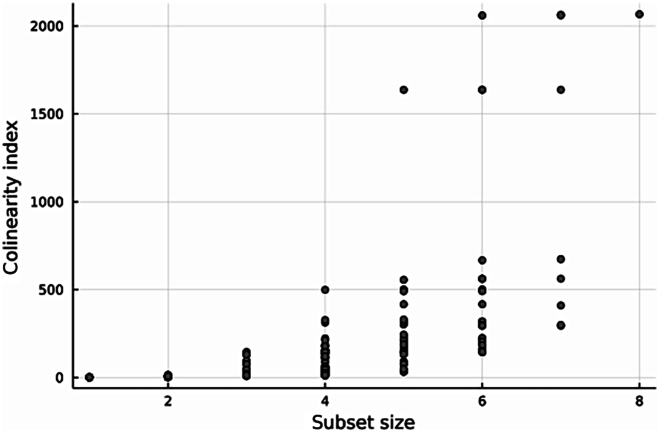
Collinearity indices for every subset, represented with respect to subset size.

Then, we computed the collinearity indexes for every subset of the parameters. Figure 2 shows that for subsets of more than three parameters, the collinearity indices can reach values larger than 500, indicating strong nonidentifiability problems. It also shows that the pairwise analysis is not sufficient: Indeed, the subsets having the highest collinearity indices all contain (*RUE*, *e_g_*, *γ*_0_, *γ_f_*, *μ_a_*), i.e., not involving the pair (*RUE*, *σ_a_*) previously studied. Such results allow determining which subsets of parameters one should avoid estimating together. We notice that adding the parameter *σ_s_* to the subset (*RUE*, *e_g_*, *γ*_0_, *γ_f_*, *μ_a_*, *σ_a_*) does not change much the collinearity index of the subset. This is also the case for *μ_s_* for the subsets (*RUE*, *e_g_*, *γ*_0_, *γ_f_*, *μ_a_*). It means that when trying to estimate the parameters of these subsets, fixing *σ_s_* or *μ_s_* will not be of much help. Thus, such a table can guide the sequencing of parameter estimation.

### Epidemic model in a plant population

As a second illustrative case study, we chose a simple model of epidemics in a plant population presented in [43]. The authors adapted the SIR epidemiological model classically used for human or animal populations: They consider a population of *N* stems that can be characterized in state *S* susceptible, *I* infected, or *R* removed, and transitions between these states are regulated by Eq. 15:

$$\left\{\begin{array}{cc} \frac{\mathrm{dS}}{\mathrm{dt}}= & b\left(\kappa -N\right)-\lambda_{0}e^{-\mu t}S\\ \frac{\mathrm{dI}}{\mathrm{dt}}= & \lambda_{0}e^{-\mu t}S\\ \frac{\mathrm{dR}}{\mathrm{dt}}= & \mathrm{dI}\\ N= & S+I+R \end{array}\right.$$ (15)

The parameters have biological values, obtained by the authors by fitting the model to their experimental data, which can be found in Table 2. These values were used as a reference point.

**Table 2.** Model parameters, their reference values, and biological meaning

*θ* ^∗^	Unit	Description	Value
b	[*time*]^−1^	Production rate of susceptible stems	1.177
*κ*	[*stems*]	Carrying capacity	4.876
*λ*_0_	[*time*]^−1^	Force of infection	0.051
*μ*	[*time*]^−1^	Decay rate of force of infection	0.096
*d*	[*time*]^−1^	Death rate of infected stems	0.246

The infected population and the total stem population are supposed to be observed every other day between days 2 and 12. We chose this observation process because (a) the data presented in [43] are measured in this way, (b) the steady state is quickly reached, and (c) we assumed that infected stems show symptoms and are therefore measurable as well as the total population. We simulated our virtual observables *y_obs_* with an additive Gaussian observation noise $\epsilon \left(t\right)∼N\left(0,\sigma^{2}\right)$, where the standard deviation *σ*^2^ is 1.5% of the typical value (i.e., the mean over time) of the corresponding observable.

#### Identifiability results

Table 3 presents the collinearity index for every parameter pair around the reference point (local values). The main offsetting effects are caused by the parameters (*μ*, *d*), with the addition of other parameters to this subset increasing the collinearity index by at most 9%.

**Table 3.** Collinearity indices for every parameter pair of the plant epidemics model. Local values around the reference vector of Table 2 and estimated mean values.

	Local values					Mean values				
par.	b	*κ*	*λ*_0_	*μ*	d	b	*κ*	*λ*_0_	*μ*	d
b	1					1				
*κ*	1.38	1				1.29	1			
*λ*_0_	1.004	1.003	1			124.4	1.003	1		
*μ*	1.002	1.002	3.30	1		81.5	1.003	1502.9	1	
d	1.002	1.002	3.41	41.70	1	52.77	1.003	583.7	175.1	1

**Fig. 3.**
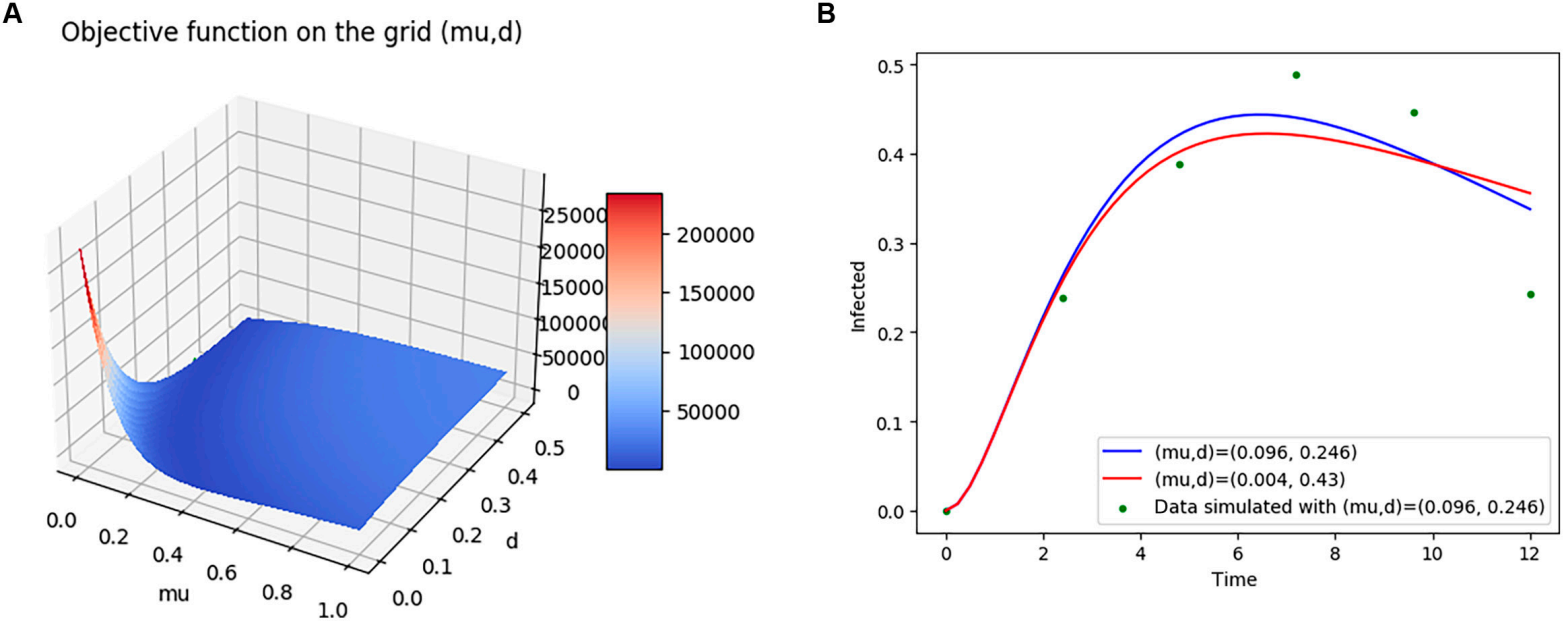
Illustration of the lack of identifiability caused by compensations between two parameters of the plant epidemics model. (A) Objective function on the grid (*μ*, *d*). (B) Dynamics of the infected for (*μ*, *d*) = (0.096,0.246) (blue curve) versus for (*μ*, *d*) = (0.004,0.43) (red), and the virtual noisy data (dots).

Figure 3 illustrates this compensation effect: It shows the associated objective function and the dynamics of the infected as a function of time for (*μ*, *d*) = (0.096,0.246) (blue curve) versus for (*μ*, *d*) = (0.004,0.43) (red curve), these values being arbitrarily chosen to be representative. Despite these parameter values being respectively 2300% and 75% different, the two curves are nearly indistinguishable, both being consistent with the data (dots).

However, these results are mainly local (around *θ*^∗^) and cannot be generalized to the entire parameter set: To investigate this fact, we calculated the collinearity indices at a different point, *θ* = (6.02,162.19,3.74,0.40,1.30), and obtained completely different results; the couple with the highest collinearity index is (*λ*_0_, *d*) with a value of 135.1 and a maximum collinearity index greater than 300 (while the maximum collinearity index at *θ*^∗^ did not exceed 50). Moreover, in practice, the identifiability analysis can be performed while the modeler has not yet acquired any experimental data and a reference point could be unavailable. The mean values of the collinearity indices are then more meaningful in that case: They are computed in Table 3, assuming $\theta ∼\mathrm{Log}-N\left(\theta^{*},1\right)$. Again, the ranking of the collinearity indices is not the same as around the reference point, showing the importance of performing the analysis on all the parameter space.

We assumed that the parameters were real values, and we computed the risk index for every parameter and the PLCI associated in Table 4. We found the highest risk index for parameters *μ*. Comparing these values between them does not really make sense as the LCIs are not of the same size; they only provide a confidence level *α* for which the parameters are practically identifiable.

**Table 4.** Profile likelihood-based confidence intervals and their associated risk index, and the ARE values

*θ* ^∗^	LCI	Risk index	sup(*α*)	ARE
b	[0.87, 7.09]	4.56	0.22	10.10
*κ*	[2.98, 97.04]	1.00	0.99	2.79
*λ*_0_	[0.001, 1.51 ]	8.00	0.13	267.62
*μ*	[-0.23, 3.70]	8.30	0.12	28.62
*d*	[-0.50, 9.05]	1.82	0.548	3.34

Finally, we computed the *ARE* for each parameter, performing each estimation from a random initial guess *θ_init_* drawn according to a log-normal distribution centered in *θ*^∗^ with standard deviation 1. We used simulated data with an additive-centered Gaussian noise with a standard deviation of 1.5% of the mean value of each observable. The ARE values are given in Table 4. The parameter *κ* has the lowest ARE (2.79), meaning it is well estimated by the procedure (Nelder–Mead algorithm). It is also the parameter with the lowest collinearity indices (Table 3) and the lowest risk index showing the consistency of all three indices. The parameters *λ*_0_ and *μ* have the greatest ARE, which is also consistent with previous results.

## Discussion

Our main objective in this study was to propose a first attempt toward a unifying framework for identifiability analysis. While not yet fully satisfying, our formalism has the advantage of bridging structural and practical identifiability through the use of the common concept of “input–output mapping” [16,44] *M*(*θ*) that we generalized and extended. Through different examples, we showed how *M* could be instantiated according to specific modeling contexts.

As regard structural identifiability, the existing definitions differed only by small details [18,19], which made this work relatively straightforward. We chose to synthesize them under the study of the injectivity of the function *θ* → *M*(*θ*). In contrast, the definitions of practical identifiability are far from consensual: Many different approaches coexist, each providing specific highlights on the identifiability problem or being applicable to specific model types [45]. We formalized the question of practical identifiability as the comparison between an estimator distribution $\hat{\vartheta }\left(y\right)$ where *y* ∼ Obs(*M*(*θ*)) for an observation protocol Obs over a model *M*(*θ*) and the distribution of an ideal (oracle) estimator. Here, again, this general framework can be specified to account for different existing indices.

More specifically, we analyzed three indices: (a) collinearity indices [32] that are a local, and partial, quantification of practical identifiability analysis since all measurements are considered as perfect (no noise), (b) profile likelihood-based confidence interval [29] that we propose to turn into a quantitative risk index instead of a binary indicator, and (c) ARE [18]. These indices allowed the detection of some compensation effects within subsets of parameters, and we confirmed that high collinearity indices corresponded to different sets of parameters producing very similar results (see Fig. 3). However, all three indices give only local information since they are computed around a reference parameter vector. We showed that this was likely to lead to unreliable results since their values could greatly vary with parameter values. We suggest that a good practice would be to average them over the whole distribution of parameter uncertainty. This seemingly straightforward solution is unfortunately hampered by the associated increase in computational needs, calling for the development of approximation methods for fast computing of these indices for large samples.

Once identifiability problems have been discovered, the natural question that arises is how to handle them. The modeler faces several options. The most drastic one is to change the model structure in order to simplify it, by removing some components or some variables [20]. This might not be wished by the modeler, especially when the model is seen as a way to formalize the current knowledge about a biological system. A second possibility would be to exploit the strength of Bayesian approaches to incorporate a priori knowledge that could better guide the estimation process toward parameter regions where the true values are more likely [20]. Third, some parameters can be set constant to their reference values, in order to reduce the dimension of the vector to estimate and limit the compensation phenomena. Parameters to set can be chosen using global sensitivity analysis [46,47] but an interesting perspective would be to combine sensitivity and identifiability indices for this purpose.

Finally, the preferential option would consist of the acquisition of additional observable variables, in order to enrich the dataset: Note that if structural nonidentifiability has been proven, it is useless to collect more data on the same variables: one has to measure new variables. The methods developed for identifiability analysis can then be reconverted into methods for the optimal design of experiments. Indeed, comparing identifiability indices for several potential candidate variables will help define which ones carry more information in terms of gain in estimation accuracy, under potential budget or time constraints [48].

A last perspective of this work would be to further explore the relationships between different indices. For instance, collinearity indices should be related to the likelihood geometry. As explained by Raue et al. [29] in the case where the functional relationship between the parameters is linear (i.e., when collinearity indices are high), a good approximation of the likelihood is computed, usually with a quadratic approximation of *χ*^2^ to the estimated optimum, e.g., the Hessian or Fisher information matrix. It would therefore make sense to make explicit these links between collinearity indices and local flatness of these *χ*^2^ approximations.

### Conclusion

This work aimed at opening new perspectives on the use of identifiability analysis for the development of plant growth models. Hopefully, it will pave the way toward an integration of the identifiability step within every estimation procedure such that, by systematically checking the uniqueness of the optimal set of parameters, we will ensure the reliability and interpretability of the parameter estimates and therefore deliver more convincing and trusted models for our applications.

## Data Availability

No experimental data were used for this study. All codes will be made available on a git repository.
